# Stochastic Boolean networks: An efficient approach to modeling gene regulatory networks

**DOI:** 10.1186/1752-0509-6-113

**Published:** 2012-08-28

**Authors:** Jinghang Liang, Jie Han

**Affiliations:** 1 Department of Electrical and Computer Engineering, University of Alberta, Edmonton, AB T6G 2V4, Canada

## Abstract

**Background:**

Various computational models have been of interest due to their use in the modelling of gene regulatory networks (GRNs). As a logical model, probabilistic Boolean networks (PBNs) consider molecular and genetic noise, so the study of PBNs provides significant insights into the understanding of the dynamics of GRNs. This will ultimately lead to advances in developing therapeutic methods that intervene in the process of disease development and progression. The applications of PBNs, however, are hindered by the complexities involved in the computation of the state transition matrix and the steady-state distribution of a PBN. For a PBN with *n* genes and *N* Boolean networks, the complexity to compute the state transition matrix is *O*(*nN*2^2*n*^) or *O*(*nN*2^*n*^) for a sparse matrix.

**Results:**

This paper presents a novel implementation of PBNs based on the notions of stochastic logic and stochastic computation. This stochastic implementation of a PBN is referred to as a stochastic Boolean network (SBN). An SBN provides an accurate and efficient simulation of a PBN without and with random gene perturbation. The state transition matrix is computed in an SBN with a complexity of *O*(*nL*2^*n*^), where *L* is a factor related to the stochastic sequence length. Since the minimum sequence length required for obtaining an evaluation accuracy approximately increases in a polynomial order with the number of genes, *n*, and the number of Boolean networks, *N*, usually increases exponentially with *n*, *L* is typically smaller than *N*, especially in a network with a large number of genes. Hence, the computational efficiency of an SBN is primarily limited by the number of genes, but not directly by the total possible number of Boolean networks. Furthermore, a time-frame expanded SBN enables an efficient analysis of the steady-state distribution of a PBN. These findings are supported by the simulation results of a simplified p53 network, several randomly generated networks and a network inferred from a T cell immune response dataset. An SBN can also implement the function of an asynchronous PBN and is potentially useful in a hybrid approach in combination with a continuous or single-molecule level stochastic model.

**Conclusions:**

Stochastic Boolean networks (SBNs) are proposed as an efficient approach to modelling gene regulatory networks (GRNs). The SBN approach is able to recover biologically-proven regulatory behaviours, such as the oscillatory dynamics of the p53-Mdm2 network and the dynamic attractors in a T cell immune response network. The proposed approach can further predict the network dynamics when the genes are under perturbation, thus providing biologically meaningful insights for a better understanding of the dynamics of GRNs. The algorithms and methods described in this paper have been implemented in Matlab packages, which are attached as Additional files.

## Background

Biological systems are inherently noisy, yet robust in the presence of noise. The function and malfunction of a system are regulated through the interactions among genes, proteins and other molecules in the cellular network. For instance, the tumour suppressor gene p53 controls cell growth and plays an important role in preventing the development and progression of tumour cells [1-4]. Therefore, it has been of great interest to understand the regulatory mechanisms of genes, and various computational models have been developed for a better understanding of gene regulatory networks (GRNs) [5].

These models can be classified into three broad categories: logical models, continuous models and stochastic models at the single-molecule level [6]. Boolean networks (BNs) are logical models that utilize discrete state levels and usually assume synchronous and discrete time steps in the evolution of a network [7], whereas continuous models, such as those using linear or ordinary differential equations [8], employ real-valued state variables over a continuous timescale. Although continuous models are in principle more accurate and may describe the dynamics of a system in more detail, they require extensive high-quality experimental data that may not always be available to modellers. As a single-molecule level model, Gillespie’s stochastic simulation algorithm (SSA) [9,10] is based on the chemical master equation; it describes the interactions among single molecules and accounts for noise and stochasticity in the regulation of a genetic network. While the SSA provides the most accurate description of the regulatory behaviour, it requires a large number of parameters and a detailed understanding of the regulatory mechanism. Despite the development of approximate SSAs that trade off accuracy for efficiency [11,12], algorithms using single-molecule level models are generally slow to run, especially in the modelling of large genetic networks.

Albeit simplistic, BNs have been shown to be efficient in the modelling of GRNs by taking advantages of low complexity and a minimum requirement on the quality (and quantity) of experimental data [13]. To account for the intrinsic noise in genetic and molecular interactions, probabilistic Boolean networks (PBNs) have been developed as a generalization of BNs [14-16]. In a PBN, the inherent stochastic nature of molecular and genetic interactions dictates that the next state of target genes is predicted by several BNs with various probabilities. The evolution of such a system is thus a Markov chain and the state transitions can be described by a transition probability matrix. A steady-state analysis further tells whether a PBN will evolve into a stable target state in the presence of random gene perturbations, thereby providing valuable information for developing intervention-based therapeutic approaches [17-21].

The computation of the steady-state distribution of a PBN, however, presents a challenge. In a PBN with *n* genes and *N* Boolean networks, the complexity to compute the state transition matrix is *O*(*nN*2^2*n*^) [15] and it is more difficult to compute the steady-state distribution. This complexity is reduced to *O*(*nN*2^*n*^) for a sparse state transition matrix [22] and can further be reduced (to the same order, but with a smaller *N*) by ignoring the Boolean networks with probabilities below certain threshold [23]. Methodologies have also been developed by eliminating genes [24] and using optimal control policies [25] to increase computational efficiency. State reduction techniques have been used for network intervention [26] and to reduce the model complexity of context-sensitive PBNs [27]. Nevertheless, it remains a difficult problem to reduce the computational complexity of a PBN without a compromise on the accuracy of an evaluation.

Although synchronicity is usually assumed in the state transitions of PBNs, asynchronous PBNs have been considered by accounting for different updating periods of genes in the constituent BNs. Asynchronous PBNs are potentially more accurate in describing the regulatory behaviour of genetic networks and may provide a better vehicle for investigating intervention strategies that lead to optimal therapeutic methodologies [28,29].

As an application of BNs, logic circuits have been used to simulate genetic networks [30]. Recently, circuit diagnosis techniques have been utilized to identify the most vulnerable molecules in cellular networks [31]. Synchronous simulation of Boolean networks has been proposed for the analysis of biological regulatory networks [32]. An unreliable logic circuit usually behaves probabilistically and thus becomes an instance of PBNs. Initially proposed for reliable circuit design [33,34], stochastic computation has been demonstrated in several physical and biological applications [35,36].

In this paper, a stochastic computational model is presented for an efficient representation and simulation of PBNs; this implementation of a PBN is referred to as a stochastic Boolean network (SBN). It is shown that in an SBN, the complexity to compute the state transition matrix is *O*(*nL*2^*n*^), where *L* is a factor related to the minimum sequence length required for obtaining an evaluation accuracy. In a network with a large number of genes, *L* is usually significantly smaller than *N*. By using a time-frame expanded structure of the SBN, the steady-state distribution can be efficiently computed. Asynchronous PBNs can also be modelled by SBNs for studying the state dynamics of GRNs. With the recent development of BN models [13,37,38], the SBN technique is potentially useful in the modelling of large genetic networks. The accuracy and efficiency of the proposed techniques are demonstrated through extensive simulation results. Albeit proposed on the formalism of PBNs, the SBN framework is potentially applicable in improving the simulation efficiency of continuous models (using linear or ordinary differential equations) and single-molecule level models such as those based on SSAs. These aspects are further discussed in the Results and Discussion section.

## Methods

### Probabilistic Boolean networks (PBNs)

In a PBN, genes are represented by a set of binary-valued nodes and the state transitions of genes are described by a list of Boolean functions. Following [15], a PBN is defined by *G (V, F)*, where *V* = {*X*_*1*_, *X*_*2*_, … *X*_*n*_}, a set of binary-valued nodes, *F =* (*F*_*1*_, *F*_*2*_, … *F*_*n*_), a list of sets of Boolean functions: $F_{i}=\left\{f_{1}^{\left(i\right)},f_{2}^{\left(i\right)},\ldots f_{l\left(i\right)}^{\left(i\right)}\right\}$ and *l*(*i*) is the number of possible functions for gene *i,*$i\in \left[1,n\right]$. Each node *X*_*i*_ represents the state of gene *i*, denoted by *x*_*i*_ and *x*_*i*_ = 1 (or 0) indicates that gene *i* is (or not) expressed. The set *F*_*i*_ contains the rules that determine the next state of gene *i*. Each $f_{j\left(i\right)}^{\left(i\right)}:{\left\{0,1\right\}}^{\text{n}}\to \left\{0,1\right\}$, for $1\leq j\left(i\right)\leq l\left(i\right)$, is a mapping or a Boolean function determining the state of gene *i*.

Due to the noise in genetic networks, the functions in a PBN occur with certain probabilities. The next state of gene *i* is determined by all the *l(i)* functions in *F*_*i*_, i.e., by $f_{1}^{\left(i\right)},f_{2}^{\left(i\right)}\ldots f_{l\left(i\right)}^{\left(i\right)}$ with probabilities $c_{1}^{\left(i\right)},c_{2}^{\left(i\right)}\ldots c_{l\left(i\right)}^{\left(i\right)}$. Thus, the next state of genes is totally determined by the possible functions and the present state of genes. This indicates that a PBN is modelled as a Markov chain. The fact that all genes are supposed to be updated synchronously also suggests a finite state machine (FSM) model for a PBN.

A PBN is independent if the functions from *F*_*i*_ are independent. This means that the selection of Boolean functions for gene *i* has no influence on the selection of Boolean functions for gene $j\left(i\neq j\right)$[39]. As a first study, the discussions in this paper are limited to independent PBNs. For an independent PBN of *n* genes, there are a total number of $N=\prod_{i=1}^{\text{n}}l\left(i\right)$ possible BNs, each of which is a possible realization of the genetic network.

For the *j*th BN $\left(1\leq j\leq N\right)$, let $f_{j}=\left[f_{j\left(1\right)}^{\left(1\right)},f_{j\left(2\right)}^{\left(2\right)}\ldots f_{j\left(n\right)}^{\left(n\right)}\right]$, where $1\leq j\left(i\right)\leq l\left(i\right)$ and *i* = 1, 2 … *n.* The probability that the *j*th BN is selected is:

(1) $$P_{j}=\prod_{i=1}^{n}c_{j\left(i\right)}^{\left(i\right)}\text{,}$$

where $c_{j\left(i\right)}^{\left(i\right)}$ is the probability that the Boolean function *j*(*i*) is selected for gene *i*. By a different selection of the BNs during a state transition, the genes can reach a different state from their present state. This property of a PBN can be described by a state transition matrix as:

(2) $$A=\left[\begin{array}{cccc} p\left(0|0\right) & p\left(1|0\right) & \ldots \ldots & p\left(\;2^{\text{n}}-1|0\right)\\ p\left(0|1\right) & p\left(1|1\right) & \ldots \ldots & p\left(\;2^{\text{n}}-1|1\right)\\ \ldots & \ldots & \ldots \ldots & \ldots \\ \ldots & \ldots & \ldots \ldots & \ldots \\ p\left(0|2^{\text{n}}-1\right) & p\left(1|2^{\text{n}}-1\right) & \ldots \ldots & p\left(2^{\text{n}}-1|2^{\text{n}}-1\right) \end{array}\right]$$

where each entry is a conditional (transition) probability that the genes transfer from a given present state into a next state. Since each BN results in a unique next state, the matrix ***A*** can be obtained by $A=\sum_{j=1}^{N}P_{j}A_{j}$, where *P*_*j*_ is the probability that the *j*th BN occurs and ***A***_***j***_ is the state transition matrix due to the *j*th BN. This way of computing ***A*** results in a complexity of *O*(*nN*2^2*n*^) [14]. Random gene perturbation, which can occur in an open genome system, is caused by random inputs from outside under external stimuli [17]. By a perturbation, a gene flips its state from 1 to 0 or vice versa. Since a PBN with perturbation is an aperiodic and irreducible homogeneous Markov chain [15], any PBN with perturbation will reach a steady state in a long run. A variant of the state transition matrix ***A*** can be used to model the effect of perturbation; however the analysis of its steady-state distribution is complex [17].

Usually, synchronicity is assumed in the state transitions of PBNs. However, a gene-level asynchronous model considers different updating periods of genes in the constituent BNs. In a deterministic-asynchronous Boolean network (DA-BN), a gene is assumed to have a fixed updating period [16]. A PBN that uses DA-BNs as constituent networks is defined as a deterministic-asynchronous probabilistic Boolean network (DA-PBN). More rigorously, a DA-PBN of *n* genes consists of a set of ${\left\{X_{i}\right\}}_{i=1}^{n}$, where *X*_*i*_ represents the expression level of the *i*th gene, denoted by *x*_*i*_ and $x_{i}\in \left\{0,1\right\}$[16]. In a DA-PBN, a gene updates its state by its updating period using the DA-BN involved. At time *t*, a binary variable *θ*_*i*_(t) can be used to indicate whether the state of gene *i* is updated or not, by a value of 1 or 0 respectively. The next state of gene *i*, *x*_*i*_(t + 1), is then determined by:

(3) $$x_{i}\left(t+1\right)=\{\begin{array}{ll} f_{j\left(i\right)}^{\left(i\right)}\left(x_{1}\left(t\right),\ldots ,x_{n}\left(t\right)\right)\;\text{with probability}\;c_{j\left(i\right)}^{\left(i\right)}, & \text{if}\;\theta_{i}\left(\text{t}+1\right)=1\\ x_{i}\left(t\right), & \text{otherwise} \end{array}$$

where $f_{j\left(i\right)}^{\left(i\right)}$ is a function in the DA-BN for gene *i*, selected with probability $c_{j\left(i\right)}^{\left(i\right)}\left(1\leq j\left(i\right)\leq l\left(i\right)\right)$.

### Stochastic Boolean networks (SBNs)

#### 1. An SBN without perturbation

In stochastic computation, real numbers are represented by random binary bit streams and information is carried in the statistics of the binary streams [40]. A stochastic processing element is typically implemented by a logic gate. Stochastic logic processes information encoded in the random binary bit streams. Probability is represented by a proportional number of bits, usually the mean number of 1’s in a bit sequence. Given independent inputs, for example, an inverter computes the complement of a probability while the multiplication of probabilities is implemented by an AND gate. Hence, stochastic computation transforms Boolean logic operations into probabilistic computation in the real domain. Signal correlations can be efficiently handled in a stochastic network by the bit-wise dependencies encoded in the random binary streams, so making it an efficient approach to computing probabilities [41].

**Figure 1:**
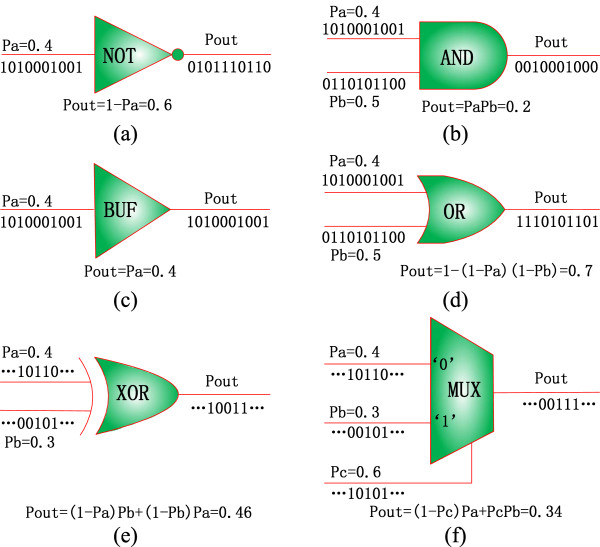
**Stochastic logic. **(**a**) a NOT gate, (**b**) an AND gate, (**c**) a buffer, (**d**) an OR gate, (**e**) an XOR gate and (**f**) a multiplexer. Stochastic logic performs arithmetic operations on the input probabilities encoded in the random binary bit streams. A probability is represented by a proportional number of bits, i.e., the mean number of 1’s in a binary sequence. For illustration, a sequence length of 10 bits is used from (a) to (d); however longer sequences are typically needed in a practical application, as shown in (e) and (f).

Figure 1 shows an inverter (NOT), an AND, a buffer, an OR, an XOR gate and a multiplexer. While an XOR gate performs a controlled inversion, a multiplexer takes one of its inputs as output according to the values of the control bits. For the 2-to-1 multiplexer of Figure 1(f), for example, its output takes the value of its input ‘a’ or ‘b’ when the control bit ‘c’ is 0 or 1. Similarly, a stochastic multiplexer chooses one of its inputs as output according to the distributions of 0’s and 1’s and thus the probability of 0 and 1 encoded in the random sequences of the control bits. For a sequence length of 1000 bits, for example, an input probability of 0.4 indicates that approximately 400 1’s are in the random sequence of the input ‘a,’ as shown in Figure 1(f). If the random input sequences are independent, the output of the multiplexer is expected to be P_a_(1 − P_c_) + P_b_P_c_ = 0.34, which means that approximately 340 1’s are expected in the output sequence. Note that this number is only approximate due to the stochastic fluctuations inherent in the representation of the random binary bit streams. This is an important feature in stochastic computation as probabilistic values are propagated rather than deterministic ones, which results in inevitable random fluctuations in the representation of probabilities. It has been shown, however, when non-Bernoulli sequences of random permutations of fixed numbers of 1’s and 0’s are used for representing initial probabilities, these fluctuations are significantly smaller than using Bernoulli sequences, which is equivalent to a random sampling based simulation [41]. It is shown later in the Result section that these fluctuations are generally negligible when reasonably long random bit sequences are used. See Additional file 1: Stochastic Logic using non-Bernoulli Sequences. Also see Additional files 2 and 3: Matlab programs that implements the functions of two-input and four-input stochastic multiplexers.

**Figure 2:**
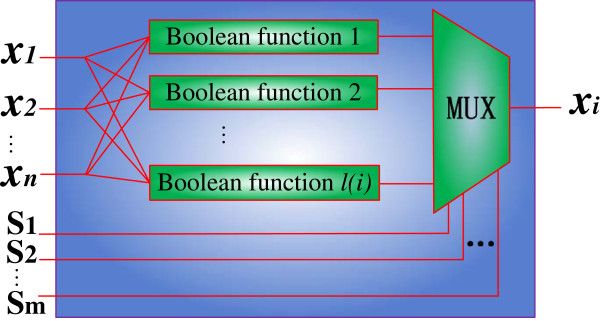
**A stochastic Boolean network (SBN) without perturbation (for a single gene).** Only the Boolean functions for a single gene *i* are shown. The control sequences ‘S_1_ ~ S_m_’ to the multiplexer (MUX) probabilistically determine the selection of Boolean functions for gene *i*. When an SBN is constructed for all the genes in a GRN, it accurately implements the probabilistic function of (1).

A general structure of the stochastic Boolean network (SBN) is defined as follows. As shown previously, the next state of genes in a PBN is updated by a set of Boolean functions according to their occurring probabilities. In an SBN, these probabilities are represented by random binary bit sequences and the selection of the Boolean functions is implemented by a stochastic multiplexer with properly generated control sequences. A general structure of an SBN for a single gene is shown in Figure 2.

Generally, if a total number of *l(i)* Boolean functions are needed to determine the next state of gene *i*, an *l(i)*-input multiplexer is used to simulate the selection of functions in the PBN for gene *i*. The number of control bits is given by $m=\left\lceil \log_{2}\left(l\left(i\right)\right)\right\rceil$. In fact, the number of possible Boolean functions for one gene is usually small—between 1 and 4 for 93% of genes [23,42]. This indicates that one or two bits are usually sufficient to control a multiplexer in an SBN. By using a stochastic multiplexer with the control bit streams S_1_ ~ S_m_, as shown in Figure 2, a function in the *j*th BN is selected with probability $c_{j\left(i\right)}^{\left(i\right)}$ for gene *i*. When all the genes are accounted for, therefore, an SBN accurately implements the probabilistic functions of a PBN, as dictated by (1).

#### 2. An SBN with perturbation

While a switch of Boolean functions may indicate a structural change in the network, a random perturbation could cause a transient change of a gene’s state under external stimuli. In a PBN with perturbation, a gene may change its value with a small probability *p* during each state transition.

Assume ***x*** = (*x*_1_, *x*_2_, … *x*_*n*_) represents the current state of an *n*-gene network at time *t* and ***γ*** is the vector that indicates the effect of random perturbation, the next state ***x***^′^ is given by [17]:

(4) $$x^{\prime }=\{\begin{array}{cc} x⊕\gamma & \text{with a probability of}\;1-{\left(1-p\right)}^{n}\\ f_{k}\left(x\right) & \text{with a probability of}\;{\left(1-p\right)}^{n} \end{array}$$

where ⊕ is the modulo 2 of additions and *f*_*k*_(·) represents the function of the *k*th Boolean network at time *t.* The effect of perturbation to the state transition matrix can then be described by a matrix called the perturbation matrix [23]. The perturbation matrix is determined by the number of genes and the gene perturbation probability *p*. It is usually computed by a (complex) analytical approach*.*

**Figure 3:**
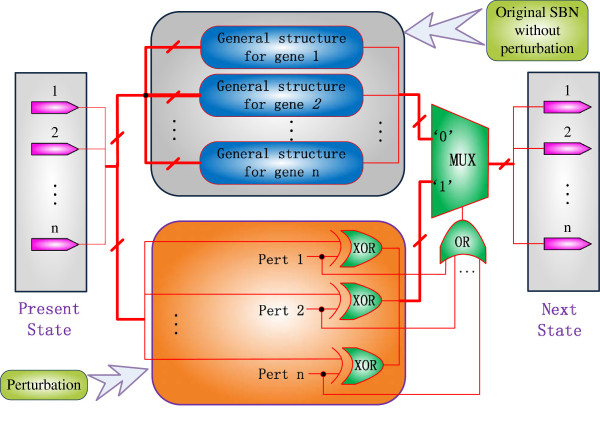
**An SBN with perturbation.** A perturbation network is implemented by the stochastic XOR logic of the perturbation vector and the present state. The probability that either a Boolean function works or a perturbation works is given by the output sequence of a stochastic *n*-input OR gate, which in turn determines the selection of a BN (without perturbation) or a perturbed network by a bus multiplexer.

However, the effect of perturbation can be readily accounted for in an SBN. Figure 3 illustrates a general model of SBNs with perturbation. As perturbation introduces a probabilistic inversion to the state of a gene, XOR gates are used to implement the addition modulo 2 of the perturbation vector and the present state. The probability that either a Boolean function works or a perturbation works (given in (4)) is computed by a stochastic *n*-input OR gate. This probability is then encoded into the output sequence of the OR gate and used as the control sequence of a bus multiplexer. If the perturbation vectors (‘Pert 1’ … ‘Pert n’ in Figure 3) are all 0’s, which means there is no perturbation, then the output sequence of the OR gate contains all 0’s, which subsequently determines that the next state is given by the original SBN without perturbation; otherwise, the next state is determined by the perturbation probability encoded in the output sequence of the stochastic OR gate. Per the stochastic functions of XOR, OR and the multiplexer, the next state is given as the output of the SBN with perturbation, by:

(5) $$x^{\prime }=\left(x⊕\gamma \right)\cdot \left(1-{\left(1-p\right)}^{n}\right)+f_{k}\left(x\right)\cdot {\left(1-p\right)}^{n}\text{,}$$

which is equivalent to (4). This indicates that a PBN with perturbation can be accurately implemented by an SBN with perturbation.

#### 3. An SBN for asynchronous PBNs

In contrast to synchronous PBNs, each gene in an asynchronous PBN has a different period of updating time. Mathematically, this is described by (3) for the so-called deterministic-asynchronous probabilistic Boolean networks (DA-PBNs). In a DA-PBN, the state of each gene is independently updated according to its own updating period.

While the deterministic asynchronicity changes the temporal sequence of state transitions, it has no impact on the logic relationships among genes, so the Boolean functions are preserved for each gene in a DA-PBN. To model this asynchronicity, an SBN can be constructed by considering the timing information as follows:

(1) Construct the Boolean functions for each gene using the proposed SBN structure.

(2) Sort the genes by the updating period and record the sequence. For example, a sequence can be created as $G_{t}=\left\{{g_{t}}^{\left(1\right)},{g_{t}}^{\left(2\right)},\ldots ,{g_{t}}^{\left(n\right)}\right\}$, where the updating periods of ${g_{t}}^{\left(1\right)},{g_{t}}^{\left(2\right)},\ldots ,{g_{t}}^{\left(n\right)}$ are in an ascending order.

(3) Consider the current first gene, i.e., the gene with the smallest updating period in *G*_*t*_, denoted by *g*_*t*_^(i)^. Since the state of *g*_*t*_^(i)^ will first be updated while the states of the other genes remain unchanged, the BNs at this stage consist of the Boolean functions of *g*_*t*_^(i)^ and buffers for the other genes. A buffer is a logic element with a delayed input as its output. In this structure, a buffer is used to preserve the state of a gene that is not being updated.

(4) Delete *g*_*t*_^(i)^ from *G*_*t*_.

(5) Repeat steps (3) and (4) until *G*_*t*_ is empty.

**Figure 4:**
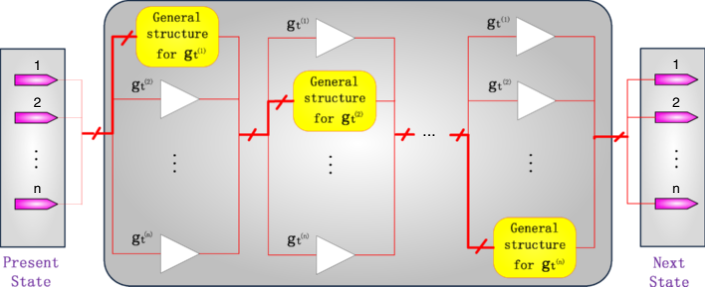
**An SBN for a deterministic asynchronous PBN.** Buffers are used to preserve the states of the genes that are not being updated.

An SBN for a DA-PBN is shown in Figure 4. Since the state transition of a fast-response gene may occur several times before a slow-response gene updates its state, the Boolean functions for a fast gene may appear in a number of times in the network of Figure 4.

### Applications of SBNs

#### 1. Computation of the state transition matrix

In an SBN, each input combination yields output sequences that contain information about the transition probability from this input state to an output state. Therefore, the statistics, i.e., the proportions of the number of each state encoded in the output sequences return the transition probabilities in a row in the state transition matrix. This row corresponds to the given input state and thus all the transition probabilities from this input can be generated in a single run. For a PBN with *n* genes, the SBN needs to be run for each of the 2^*n*^ input states and an *O*(*n*) number of sequences need to be generated for the control signals of the multiplexers.

The accuracy in the computed state transition probabilities is determined by the sequence length of the random binary bit streams. In general, longer sequences are required in a larger network for achieving an evaluation accuracy. To consider the overhead incurred in the use of a larger sequence length, a factor, *L*, is introduced and therefore, a complexity of *O*(*nL*2^*n*^) results for computing all the entries in the state transition matrix for a desired accuracy.

It has been shown that the required sequence length is related to the reliability and thus the size of a combinational network [41]. In an SBN, the network size is typically on a polynomial order of the number of genes. This is in contrast with the number of BNs, *N*, which generally increases exponentially with the number of genes. As a result, the complexity of using an SBN to compute the transition matrix, i.e., *O*(*nL*2^*n*^), is significantly smaller than the analytical result of *O*(*nN*2^*n*^), especially for a network with a large number of genes. This is demonstrated later by simulations using several measures to determine the minimum sequence length required for certain accuracy.

The procedure of computing the state transition matrix using an SBN is summarized as follows:

(1) Construct an SBN by inserting a multiplexer for each gene in a PBN;

(2) For each input state, generate initial random binary streams encoding the control signal probabilities for each multiplexer;

(3) Propagate the binary streams from the present state (inputs) to the next state (outputs) and obtain a random bit sequence for each output;

(4) Obtain the statistics, i.e., the proportions of the number of each state encoded in the output sequences as the transition probabilities for this input state;

(5) Repeat steps (2), (3) and (4) for all 2^n^ input states to compute all the entries in the state transition matrix.

For an SBN with perturbation, the state transition matrix can be similarly computed using the procedure outlined above with an exception in the construction of the SBN in step (1).

#### 2. Estimation of the steady-state distribution

**Figure 5:**
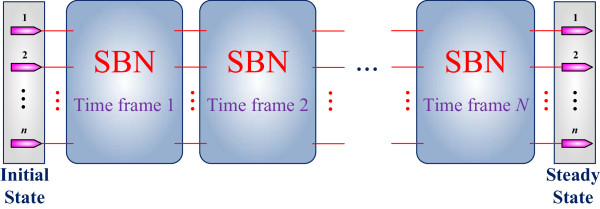
**A time-frame extended SBN.** Each module is an implementation of the original SBN without or with perturbation (as in Figure 3). A time-frame extended SBN can be used for an efficient analysis of the steady-state distribution of a GRN under perturbation.

Given the size of the state transition matrix of a PBN, the analysis of the steady-state distribution is challenging for using both analytical and simulative approaches. The Markovian nature of a PBN makes its analysis similar to that of a finite state machine (FSM). An FSM is equivalent to a sequential circuit implementation. By a time-frame expansion, a sequential circuit can be unrolled into a series of identical combinational modules connected in the spatial domain. Using a similar technique, the temporal operation of an SBN can be transformed into a spatial operation of identical SBNs connected in series. This is shown in Figure 5. This spatial extension of an SBN can be used for the steady-state analysis and the required iterations of the SBN are determined by the number of state transitions before reaching a steady state.

A steady-state analysis using a time-frame expanded SBN starts with an initial input state, generates the random bit sequences for the inputs and control bits of multiplexers, and then propagates the stochastic signals through the expanded SBN structure. This process is equivalent to an analytical procedure of multiplying the input probabilities with the powers of the state transition matrix. Finally, a small variance threshold is used to determine whether the system has reached a steady state. The steady-state distribution is then obtained from the output sequences at the end of the operation.

In the above process, the speed of convergence to a steady state is dependent on a number of factors, including the length of random bit sequences, the variance threshold value and the perturbation rate. In practice, a sequence length that is long enough to have a resolution of at least two magnitudes smaller than the threshold value, is used to guarantee that the convergence is not dominated by stochastic fluctuations. It is shown later that the analysis using an extended SBN structure provides an alternative and efficient way of estimating the steady-state distribution of a PBN without resorting to the state transition matrix.

### Example: the p53-Mdm2 network

**Figure 6:**
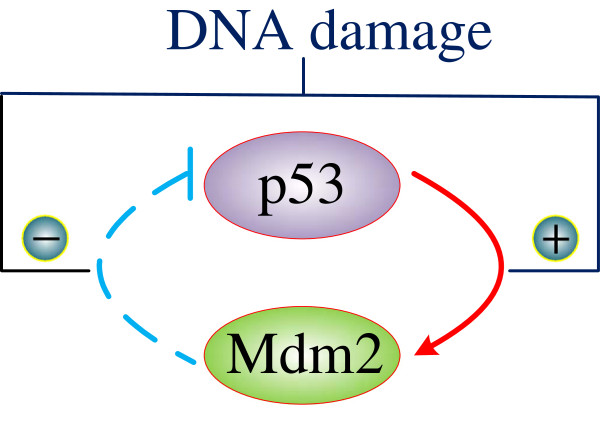
**The p53-Mdm2 network (adapted from **[3]**).** Under DNA damage, p53 promotes the expression of the Mdm2 gene, which in turn causes the degradation and destruction of p53.

In a p53 network, signaling pathways are triggered by DNA damages and external factors such as chemotherapeutic drugs and ultraviolet light. For instance, DNA double strand breaks (DSBs) activate pathways that involve the p53 and Mdm2 genes (Figure 6) [3,4]. In response to DSBs, the ATM kinase is first stimulated and the Chk2 is then stimulated by ATM. These activated kinases subsequently induce an increase in the concentration level of p53 and a decrease in the interactions between p53 and Mdm2. The increase in the p53 protein level and its transcription activity promote the expression of the Mdm2 gene, which in turn proceeds to trigger the degradation and destruction of p53. This prior knowledge enables us to come up with the transition rules for the p53-Mdm2 interactions, as shown in Table 1. Based on these rules, an independent PBN of the two genes p53 and Mdm2 can be established: V = (*X*_1_, *X*_2_) with the function classes ${\text{F}}_{1}=\left\{f_{1}^{\left(1\right)},f_{2}^{\left(1\right)},f_{3}^{\left(1\right)},f_{4}^{\left(1\right)}\right\}$ and ${\text{F}}_{2}=\left\{f_{1}^{\left(2\right)},f_{2}^{\left(2\right)},f_{3}^{\left(2\right)},f_{4}^{\left(2\right)}\right\}$. The state transitions of this PBN are given in the truth table of Table 2.

**Table 1:** State transition probabilities of the p53-Mdm2 network

**Present State**	**Next State Probability**			
**p53, Mdm2**	**p53**		**Mdm2**	
(or, ***x***_**1**_***x***_**2**_)	0	1	0	1
00	0.01	0.99	0.99	0.01
01	0.1	0.9	0.9	0.1
10	0.9	0.1	0.1	0.9
11	0.5	0.5	0.5	0.5

**Table 2:** Truth table of the PBN for the p53-Mdm2 network

**x**_**1**_**x**_**2**_	$f_{1}^{\left(1\right)}$	$f_{2}^{\left(1\right)}$	$f_{3}^{\left(1\right)}$	$f_{4}^{\left(1\right)}$	$f_{1}^{\left(2\right)}$	$f_{2}^{\left(2\right)}$	$f_{3}^{\left(2\right)}$	$f_{4}^{\left(2\right)}$
00	1	1	1	0	0	0	0	1
01	1	1	0	0	0	0	1	1
10	0	0	1	1	1	1	0	0
11	0	1	1	1	1	0	0	0
$c_{j}^{\left(i\right)}$	0.5	0.4	0.09	0.01	0.5	0.4	0.09	0.01

In Table 2, the leftmost column indicates the present state of the genes p53 and Mdm2. The internal entries in the table indicate whether a function will result in a logical 1 or 0 at the next state of each gene. The row on the bottom shows the probability of each transition by a function. Given an initial state of ‘01,’ for example, the next state of the genes can be ‘00’ with a probability of (0.09 + 0.01) × (0.5 + 0.4) = 0.09, ‘01’ with a probability of (0.09 + 0.01) × (0.09 + 0.01) = 0.01, ‘10’ with a probability of (0.5 + 0.4) × (0.5 + 0.4) = 0.81 or ‘11’ with a probability of (0.5 + 0.4) × (0.09 + 0.01) = 0.09. A PBN is determined by the truth table of Table 2 and its state transition matrix can be computed as:

(6) $$A_{\mathrm{PBN}}=\left[\begin{array}{llll} 0.0099 & 0.0001 & 0.9801 & 0.0099\\ 0.0900 & 0.0100 & 0.8100 & 0.0900\\ 0.0900 & 0.8100 & 0.0100 & 0.0900\\ 0.2500 & 0.2500 & 0.2500 & 0.2500 \end{array}\right]\text{.}$$

**Figure 7:**
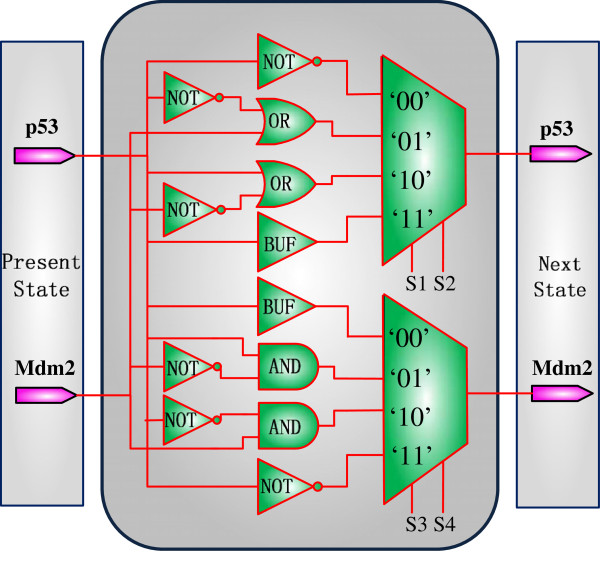
**An SBN for the p53-Mdm2 network (without perturbation).** This SBN implements the truth table of the PBN defined in Table 2 for the network in Figure 6. It has 2 genes and 16 Boolean networks in total.

For this PBN, an SBN can be constructed using stochastic multiplexers and random binary bit streams as information carriers, as shown in Figure 7. As discussed previously, the control binary sequences determine the probability that each Boolean network is selected. For example, as the Boolean functions for the p53 gene occur with probabilities 0.5, 0.4, 0.09 and 0.01, the binary bit sequences for the control vectors ‘S1S2’ to the multiplexer are generated with a probability of 0.5 to be ‘00,’ a probability of 0.4 to be ‘01’, a probability of 0.09 to be ‘10’ and a probability of 0.01 to be ‘11.’ Then the output bit sequences are read out and decoded into (transition) probabilities. With a sequence length of 10000 bits, the state transition matrix is obtained as follows:

(7) $$A_{\mathrm{SBN}}=\left[\begin{array}{llll} 0.0097 & 0.0003 & 0.9803 & 0.0097\\ 0.0899 & 0.0101 & 0.8101 & 0.0899\\ 0.0904 & 0.8096 & 0.0096 & 0.0904\\ 0.2511 & 0.2489 & 0.2489 & 0.2511 \end{array}\right]\text{.}$$

The difference between (6) and (7) is evaluated using the following norms: ${\left\|.\right\|}_{1}$ and ${\left\|.\right\|}_{\infty }$, which specify the maximum absolute value of the summed differences of columns and rows of the two matrices respectively, and ${\left\|.\right\|}_{2}$, which is a measure on the average difference of all the entries in these matrices. For (6) and (7), we obtain $\left\|{\overset{}{A}}_{\mathrm{SBN}},-,{\overset{}{A}}_{\mathrm{PBN}}\right\|{}_{1}=0.0018$, $\left\|{\overset{}{A}}_{\mathrm{SBN}},-,{\overset{}{A}}_{\mathrm{PBN}}\right\|{}_{2}=0.0024$ and $\left\|{\overset{}{A}}_{\mathrm{SBN}},-,{\overset{}{A}}_{\mathrm{PBN}}\right\|{}_{\infty }=0.0044$, which indicate that the SBN structure accurately computes the state transition matrix of the PBN.

**Figure 8:**
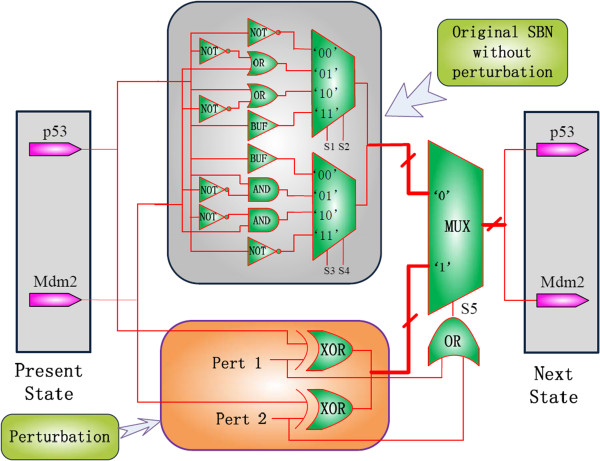
An SBN for the p53-Mdm2 network (with perturbation).

With random gene perturbation, an SBN with perturbation can be constructed, as shown in Figure 8. If the stochastic OR outputs a ‘1’ (indicated by S5 in Figure 8), which means that at least one of the p53 and Mdm2 are perturbed, the multiplexer is then switched to the perturbation network. If the output of the OR is 0, the multiplexer is switched to the original SBN and the network works as the one in Figure 7 without perturbation.

A similar procedure can be used to compute the state transition matrix of the SBN with perturbation—the result is shown in (8) for a perturbation probability of 0.01:

(8) $${\tilde{A}}_{\mathrm{SBN}}=\left[\begin{array}{llll} 0.0097 & 0.0100 & 0.9705 & 0.0098\\ 0.0975 & 0.0106 & 0.7946 & 0.0973\\ 0.0998 & 0.7921 & 0.0082 & 0.0999\\ 0.2444 & 0.2565 & 0.2551 & 0.2440 \end{array}\right]\text{.}$$

Compared to the analytical result by a method based on (4):

(9) $${\tilde{A}}_{\mathrm{PBN}}=\left[\begin{array}{llll} 0.0097 & 0.0100 & 0.9705 & 0.0098\\ 0.0981 & 0.0098 & 0.7940 & 0.0981\\ 0.0981 & 0.7940 & 0.0098 & 0.0981\\ 0.2451 & 0.2549 & 0.2549 & 0.2450 \end{array}\right]\text{,}$$

the differences between (8) and (9) are revealed in the measures of $\left\|{\tilde{A}}_{\mathrm{SBN}},-,{\tilde{A}}_{\mathrm{PBN}}\right\|{}_{1}=0.0032$, ${\left\|{\tilde{A}}_{\mathrm{SBN}},-,{\tilde{A}}_{\mathrm{PBN}}\right\|}_{2}=0.0030$ and $\left\|{\tilde{A}}_{\mathrm{SBN}},-,{\tilde{A}}_{\mathrm{PBN}}\right\|{}_{\infty }=0.0042$. This shows that the proposed approach using an SBN can accurately and efficiently compute the state transition matrix. The differences in these results come from the stochastic fluctuation, which is an intrinsic property of stochastic computation. More simulation results are presented in the Results and Discussion section, which show that the fluctuations are generally small. A steady state analysis using (8) further confirms the p53-Mdm2 oscillatory dynamics observed in experiments.

An SBN for an asynchronous p53-Mdm2 network can also be constructed, as in Figure 4 and following the aforementioned procedure. Due to space limitation, however, this is not further discussed and will be pursued in future work.

## Results and discussion

### Simulations with randomly generated networks

The state transition matrices of several randomly generated PBNs have been computed using the proposed SBN structure. The Boolean functions of each network are generated for a given number of genes (*n*) and a total number of BNs (*N*). The simulation is run on a PC with an Intel Core i3-2100 CPU (@3.10 GHz) and 6G memory. The results for using sequence lengths of 10000 and 1000 bits are first compared to those obtained using an analytical approach, as shown in Table 3. While a larger sequence length of 10000 bits produces results with a higher precision, a sequence length of 1000 bits also provides highly accurate results for networks of such size.

**Table 3:** **Errors in the state transition matrices obtained using SBNs without perturbation, compared to the results by using the analytical approach in**[22]

**Number of genes (*n*)**		**2**	**3**	**4**	**5**	**6**
**Error**	**Length (bits)**					
${\text{Error}}_{{\left\|\cdot \right\|}_{1}}$	1000	0.0070	0.0330	0.0420	0.0477	0.0649
	10000	0.0027	0.0052	0.0105	0.0179	0.0186
${\text{Error}}_{{\left\|\cdot \right\|}_{2}}$	1000	0.0100	0.0314	0.0408	0.0287	0.0405
	10000	0.0038	0.0047	0.0102	0.0109	0.0099
${\text{Error}}_{{\left\|\cdot \right\|}_{\infty }}$	1000	0.0160	0.0640	0.0908	0.0735	0.1293
	10000	0.0056	0.0096	0.0248	0.0303	0.0248

In general, a smaller sequence length leads to a shorter run time in the computation of state transition matrices. However, the error incurred due to stochastic fluctuations increases with the size of the network under evaluation. Subsequently, therefore, a minimum accuracy requirement is given and the length of the stochastic sequence is increased for a larger network in order to meet this requirement. Tables 4 and 5 show the minimum sequence lengths and run time required for two different accuracy values, given by the aforementioned “Norm 2” that measures the average difference of all the entries in two matrices. In this experiment, networks of various sizes with up to 12 genes are considered. For each size, five random networks are generated as follows. Given the number of genes in a network, the number of Boolean functions for each gene is initially randomly determined; the specific functions and their associated probabilities are then randomly generated; finally, the input genes are randomly selected for each function. Since a gene’s state is usually determined by no more than four Boolean functions [42], the number of Boolean functions is considered no larger than 4 for each gene. For simplicity, each Boolean function is selected from a set of basic functions: the buffer, NOT, AND, NAND, OR, NOR, XOR and XNOR. In this process, pseudo-random numbers are generated and used in the random selections. For these networks, the standard deviations of the minimum sequence lengths and run time are also shown in Tables 4 and 5. It can be seen that the SBN approach requires a significantly shorter runtime than the analytical approach, especially in the evaluation of large networks. Next, the efficiency of the SBN technique is compared to that of an approximate analytical approach [23] for several networks with more than 10 genes. The results are shown in Table 6.

**Table 4:** Minimum sequence length and run time required in the computation of state transition matrices for a given accuracy, measured by Norm 2

**n**	**N**	**SBN (Norm 2 = 0.04)**				**SBN (Norm 2 = 0.02)**				**Method**[22]	
		**Sequence length**	**Std. deviation**	**Avg. time (s)**	**Std. deviation**	**Sequence length**	**Std. deviation**	**Avg. time (s)**	**Std. deviation**	**Avg. time (s)**	**Std. deviation**
2	6	150	46	0.006324	0.003315	480	84	0.013655	0.007568	0.005468	0.004100
3	8	460	89	0.019755	0.008942	800	122	0.017634	0.009536	0.011655	0.007036
4	16	520	109	0.024337	0.009108	1120	84	0.043844	0.010102	0.031391	0.009388
5	32	860	134	0.052112	0.017356	1540	182	0.118927	0.036943	0.157794	0.020922
6	64	1240	270	0.209416	0.030298	2460	241	0.548156	0.042366	0.532971	0.037483
7	128	1340	167	0.453192	0.048960	3680	239	1.208252	0.060325	2.441066	0.163347
8	256	2260	378	2.030217	0.171125	5480	335	4.110083	0.326308	9.368184	0.863544
9	512	2580	303	4.751360	0.421918	6820	471	12.81050	2.061854	39. 26049	4.208466
10	1024	3920	923	16.06112	4.252810	8760	1135	38.60258	6.377620	201.5433	10.90932
11	2048	4700	836	40.44380	5.742303	10400	1140	95.40610	7.547263	811.6358	15.88395
12	4096	5660	882	118.3426	9.031772	13000	1000	286.5043	12.37633	3501.744	86.66141

(no perturbation, *n*: the number of genes, and *N*: the number of BNs). The results are obtained from five randomly generated networks, so the standard deviations of the minimum sequence length and run time are also shown.

**Table 5:** Minimum sequence length and run time required in the computation of state transition matrices for a given accuracy, measured by Norm 2

**n**	**N**	**SBN (Norm 2 = 0.04)**				**SBN (Norm 2 = 0.02)**				**Method**[22]	
		**Sequence length**	**Std. deviation**	**Avg. time (s)**	**Std. deviation**	**Sequence length**	**Std. deviation**	**Avg. time (s)**	**Std. deviation**	**Avg. time (s)**	**Std. deviation**
2	6	180	45	0.008052	0.005219	340	55	0.017285	0.010683	0.050477	0.010140
3	8	460	114	0.020473	0.011034	920	130	0.027358	0.010944	0.026389	0.014326
4	16	660	152	0.032089	0.023041	1220	148	0.055602	0.022138	0.053726	0.021034
5	32	880	130	0.071256	0.020862	1620	130	0.162794	0.047719	0.161462	0.039981
6	64	1320	228	0.235628	0.038845	2460	288	0.443522	0.056302	0.613840	0.047252
7	128	1480	130	0.574352	0.062129	4240	261	1.540875	0.071316	2.663523	0.180211
8	256	2420	319	2.124709	0.228612	5620	319	4.411751	0.413352	11.90834	1.412206
9	512	3220	650	7.248265	2.301722	6940	498	14.36077	3.253704	61.45203	6.881528
10	1024	4140	882	18.09032	4.112405	9400	1140	41.41356	5.289815	261.3189	12.29343
11	2048	4860	606	47.37403	5.822926	11800	837	120.3839	6.107372	975.3821	33.25207
12	4096	5820	782	132.8137	10.90686	14600	1342	373.7601	13.64551	4022.140	78.42531

(perturbation probability = 0.01, *n*: the number of genes, and *N*: the number of BNs). The results are obtained from five randomly generated networks, so the standard deviations of the minimum sequence length and run time are also shown.

**Table 6:** **Run time and errors in the computation of state transition matrices for SBN and the approximate method in**[23]

**n**	**N**	**SBN (s) (Length = 10000 bits)**	**Method**[23]**(s) (lower bound = 10**^**-4**^**)**	${\text{Error}}_{{\left\|\cdot \right\|}_{2}}$**(SBN)**			${\text{Error}}_{{\left\|\cdot \right\|}_{2}}$[23]		
				${\text{Error}}_{{\left\|\cdot \right\|}_{1}}$	${\text{Error}}_{{\left\|\cdot \right\|}_{2}}$	${\text{Error}}_{{\left\|\cdot \right\|}_{\infty }}$	${\text{Error}}_{{\left\|\cdot \right\|}_{1}}$	${\text{Error}}_{{\left\|\cdot \right\|}_{2}}$	${\text{Error}}_{{\left\|\cdot \right\|}_{\infty }}$
11	2048	92.367577	183.617225	0.2031	0.0268	0.1209	0.2416	0.0463	0.0221
12	4096	221.849183	1125.969347	0.3448	0.0301	0.1540	0.6387	0.0929	0.0386
13	8192	489.265478	4395.954714	0.4581	0.0552	0.2249	1.6583	0.1414	0.0874
14	16384	1063.892415	9415.812415	1.0152	0.0825	0.4287	2.1642	0.2283	0.1895

(no perturbation, *n*: the number of genes, and *N*: the number of BNs).

As revealed in the tables, while an analytical approach is fast in computing the state transition matrices of small networks, it becomes cumbersome to use for larger networks. This is because an analytical approach is limited by the number of BNs (*N*), which generally increases exponentially with the number of genes in a PBN. In an SBN, however, all the state transition probabilities for each input state are encoded in the output sequences, so the computation of the state transition matrix is very efficient. Although a longer stochastic sequence length is required to meet a higher accuracy, the proposed SBN approach still outperforms an analytical approach for networks with a large number of genes and BNs, because its efficiency is not directly limited by the number of BNs.

The state transition matrix computed using an SBN can be used to obtain the steady state distribution of a network. However, the size of the network that can be evaluated is restricted due to the exponential increase of the size of the matrix. As an alternative and efficient approach, the time-frame expansion technique can be used to evaluate much larger networks under perturbation. Recently, several BN models have been developed for GRNs with tens of genes [13,37,38]. Although the parameters for use in a PBN have not been obtained, the time frame expansion technique is well suited for simulating a network of such size, once the necessary parameters become available. In Table 7, the average runtime for simulating networks of 20 and 30 genes is shown for various accuracy requirements and perturbation rates. Since the runtime for reaching the steady state is dependent on the initial probabilities (as applicable in the general Markov chain theory), five independent experiments with randomly-selected initial probabilities are performed to obtain an average result. However, it should be noted that the run time of the time-frame expanded SBN technique is also dependent on the threshold value and perturbation rate. In Table 7, therefore, the average number of periods and run time for convergence, as well as their standard deviations, are shown for several threshold values and perturbation rates. It can be seen, for example, that a 20-gene network with a perturbation rate of 0.01 can be evaluated in approximately 2.6 seconds using the time-frame expanded SBN technique for a threshold value of 0.01 (Norm infinity). These results indicate that the time-frame expanded SBN technique is potentially useful in the analysis of large GRNs.

**Table 7:** Run time of the time frame expansion technique for randomly-generated networks

**Number of genes**	**Sequence length (bits)**	**Threshold value (Norm infinity)**	**Perturbation rate**	**SBN (results from five experiments with different initial values)**			
				**Average number of periods for convergence**	**Standard deviation of the number of periods**	**Run time (s)**	**Standard deviation of the run time (s)**
20	100,000	0.001	0.0001	1648	278.3	1477.8	125.1
	100,000	0.001	0.001	202	21.3	182.65	18.62
	10,000	0.01	0.01	20	4.9	2.5878	0.5663
30	1,000,000	0.01	0.0001	1128	173.2	15536	3121
	1,000,000	0.1	0.001	66	26.2	904.04	395.6

### Experiments on a T-cell time series dataset

A network inferred from a time series gene expression dataset [43] is further modelled using SBNs. The dataset was taken from an IL-2-stimulated immune response experiment using a murine T cell line called CTLL-2. Cells were collected at 12 different time points before IL-2 stimulation (0 h) and after IL-2 stimulation (15, 30 mins, 1, 2, 4, 6, 8, 10, 12, 16 and 24 h). The dataset was then normalized to the same expression level and clustered based on the similarities in the regulatory behaviour of the genes. This produced simplified networks of gene groups, referred to as meta-genes, instead of actual genes. This result has significantly reduced the complexity of the analysis and interpretation of the inferred networks. Finally, the dataset was discretized for the implementation of a Boolean network inference algorithm [43]. This algorithm is discussed in detail next.

#### 1. Inference of Boolean dynamics of the GRN

PBNs have been inferred from steady-state data using the coefficient of determination [15] and from time series data to estimate the perturbation probabilities and switching probabilities between the constituent BNs [44]. Large amounts of data are usually required by these methods due to their computational complexity. In [43], the Boolean inference is based on the activation and inhibition functions of a target gene and its control genes. This is similar to the qualitative inference method used in [45], but it considers all possible networks rather than a single most likely one. While the number of possible inputs to a Boolean function is limited in this method, the restriction on the amount of data required to perform an inference is released. The number of possible networks is then counted and all networks are enumerated.

**Figure 9:**
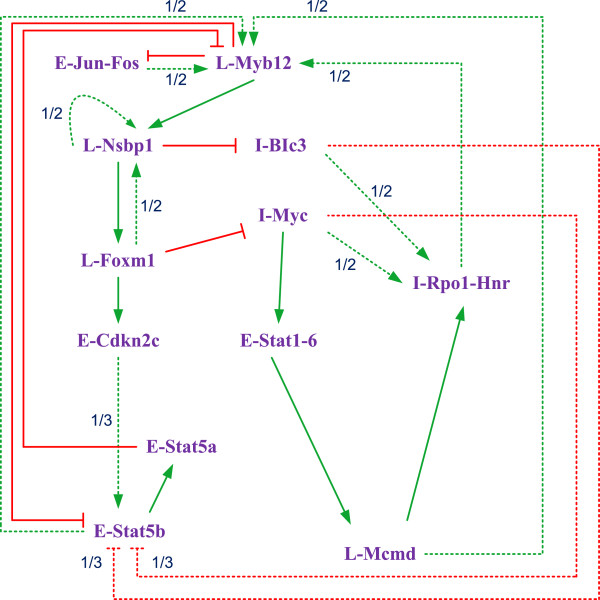
**A T cell immune response network inferred from a time series gene expression dataset (adapted from **[43]**).** Solid arrows indicate relationships occurring in all of the 901 networks, while the numbers associated with the dashed arrows indicate the fraction of networks having that relationship. The green lines represent activation relationships and the red lines represent inhibition relationships.

For the T-cell time series dataset, a total of 161,558 networks were discovered by the inference algorithm [43]. The inference algorithm further explores the dynamics of the inferred networks. This is based on the fact that finite BNs are expected to exhibit a cyclic pattern of expression [7]. During this step, the steady states or attractors are computed to validate the inferred networks. It was found that 160,657 (99.4%) of these networks did not exhibit the fluctuations expected in the steady-state dynamics of the IL-2 stimulated T cell network [43]. Therefore, these networks were discarded and 901 (0.6%) of the networks that produced biologically meaningful attractors were left for further analysis. The 901 networks were based on twelve meta-genes and yielded a consensus network as shown in Figure 9. The steady-state dynamics in the 901 networks consist of three time points (shown in Table 3 of [43]). It has also been shown that the computational complexity of this inference algorithm increases exponentially with the maximum number of inputs to a node [43]. However, the maximum input number is limited by the size of a network with a power law [46], so this number is expected to be smaller than 5 for a network with less than 100 nodes.

The resulting network is not unique in that the occurrence of different Boolean functions results in different BNs. In Figure 9, the activation and inhibition relationships that occur in all 901 networks are indicated by solid arrows, while the relationships that occur in a fraction of the networks are indicated by dashed arrows. The value associated with a dashed arrow indicates the fraction of networks having that relationship. To infer a PBN, this fractional occurrence of a function is considered probabilistic and its associated value is taken as the occurrence probability of a Boolean function in the network. These probabilities are then utilized to obtain the switching probabilities between the constituent BNs in the PBN. Since a solid arrow indicates a relationship that exists in all 901 networks in Figure 9, this function is considered to occur with a probability of 1. The inferred PBN is shown in the truth tables (see Additional file 4: Truth table of the PBN inferred from the T cell microarray time series data), for which the Boolean functions are assumed to occur independently in a BN.

#### 2. Modeling the network with SBN

To build an SBN for the inferred network of Figure 9, each of the 12 genes is assigned a number, as shown in Table 8. For these 12 genes, there are 2^12^ or 4096 states, each of which is indexed by the state of each gene as follows:

(10) $$k=\sum_{i=1}^{12}g\left(i\right)\cdot 2^{i-1}+1\text{,}$$

where *i* is the gene index and *g(i)* is the state of gene *i* (i.e., 1 or 0).

**Table 8:** Code of the 12 genes in the T cell immune response network

**Gene**	**E-Jun-Fos**	**L-Nsbp1**	**L-Foxm1**	**I-BIc3**	**I-Myc**	**L-Myb12**	**E-Cdkn2c**	**E-Stat1-6**	**I-Rpol-hnr**	**E-stat5a**	**E-stat5b**	**L-Mcmd**
Symbol	g(1)	g(2)	g(3)	g(4)	g(5)	g(6)	g(7)	g(8)	g(9)	g(10)	g(11)	g(12)

Since solid arrows in Figure 9 indicate regulatory interactions found in all 901 networks, they are considered to have a priority over other interactions, i.e., any other relationships are overruled by a solid-line interaction if they occur simultaneously. For the dashed arrows, the priority is determined according to the observations in the experiments. Take ‘E-stat5b’ for example; the solid arrow indicates that L-Myb12 inhibits E-stat5b in all the networks, so the activation of L-Myb12 overrules any other function applied on E-stat5b. When the state of E-stat5b is only affected by the dashed arrows, the activation by E-Cdkn2c is considered to take precedence over the inhibitions by I-BIc3 and I-Myc, as the upregulation of E-stat5b has been observed in the experiments.

**Figure 10:**
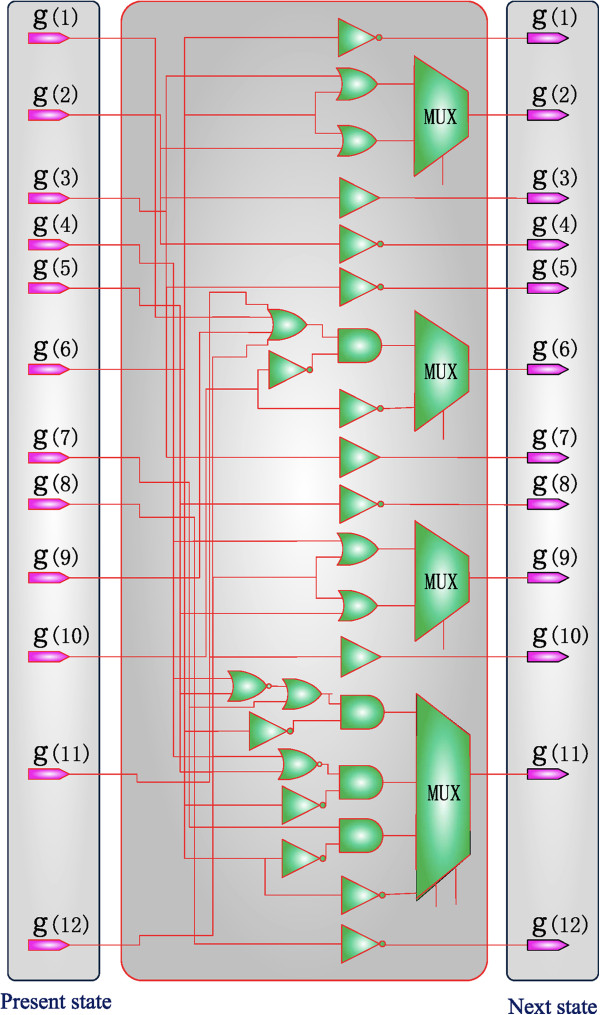
**An SBN for the GRN in Figure**9.

An SBN is constructed for the genetic network of Figure 9, as shown in Figure 10. The construction is based on the following principles:

(1) An inhibited signal is considered logical “low” while an activated signal is considered logical “high.” Therefore, an inverter or a buffer is applied to represent an inhibition or an activation relationship between genes. For example, L-Myb12 inhibits E-Jun-Fos, so an inverter is used to simulate this relationship between *g*_*t*_(6) and *g*_*t+1*_(1). For the activation of L-Foxm1 by L-Nsbp1, a buffer is applied between *g*_*t*_(2) and *g*_*t+1*_(3).

(2) An OR gate is applied to model multiple activations while a NOR (inverted OR) gate is applied to model multiple inhibitions on the same gene. For example, L-Myb12 can be activated by any one of E-Jun-Fos, I-Rpol-Hnr, E-stat5b and L-Mcmd, so in Figure 10, *g*_*t*_(1), *g*_*t*_(9), *g*_*t*_(11) and *g*_*t*_(12) are used as the four inputs to an OR gate. However, due to the inhibition of L-Myb12 by E-stat5a, an inverter is applied and its output is ANDed with the output of the 4-input OR gate to produce the output of *g*_*t+1*_(6). The use of the AND is dictated by the priority rule of the inhibition over the activation of L-Myb12, as explained as follows.

(3) When an inhibition and activation occur on the same gene, the logic gate is determined by the priority of the two functions: an AND gate is applied if the inhibition has a higher priority, whereas an OR gate is used if the activation has a higher priority. For instance, an AND gate is used to model the relationship between the activation and inhibition of L-Myb12 in the example of (2), as shown in Figure 10.

(4) A solid arrow indicates a relationship that exists in all 901 networks and therefore is considered to occur with a probability of 1. The corresponding function then exists in every Boolean function that produces an input to a MUX. For example, E-stat5a inhibits L-Myb12 in all the networks, so inverters are present in both of the two Boolean functions that lead to *g*_*t+1*_(6).

#### 3. Steady-state evaluation

For this SBN, the state transition matrix ***A***_*T*_ is of the size 4096 x 4096 and computed in about 70s. See Additional file 5: The Matlab program that describes the structure of the SBN in Figure 10 and computes its state transition matrix (for both without and with perturbation).

Given an initial input, ***I***_***0***_ = [0, 0… 0, 1, 0… 0], as indicated by the vector at *T = 1 h* in Table 3 of [43] that corresponds to the state 1730 (by (10)), the output response after *t* clock cycles can be computed by:

(11) $$\text{Output}\left(t\right)=I_{0}A_{T}^{t}$$

A clock cycle here corresponds to the time interval between two discrete time points as a period of biological response. It has been shown that the network exhibits a steady-state dynamics consisting of three time points [43]. Although these steady states, or attractors, can be computed using a BN-based method (e.g. [47]), (11) is used here to estimate the attractors as a means to validate the constructed T-cell SBN. In this evaluation, a periodic behaviour of state transitions has been observed after 20 clock cycles.

As shown in Table 9, the obtained stationary states with the highest probabilities perfectly match the three attractors found at the time points *t1*, *t2* and *t3* in [43], referred to as Attractors 1, 2 and 3 at states 1224, 1768 and 711.

**Table 9:** **Attractors found by the SBN approach, compared to the experimental results in**[43]

**Number of cycles**	**States with highest probabilities**	**Attractors found in**[43]
28	1224	Attractor 1
	711	Attractor 3
	1768	Attractor 2
29	1768	Attractor 2
	1224	Attractor 1
	711	Attractor 3
30	711	Attractor 3
	1768	Attractor 2
	1224	Attractor 1

**Figure 11:**
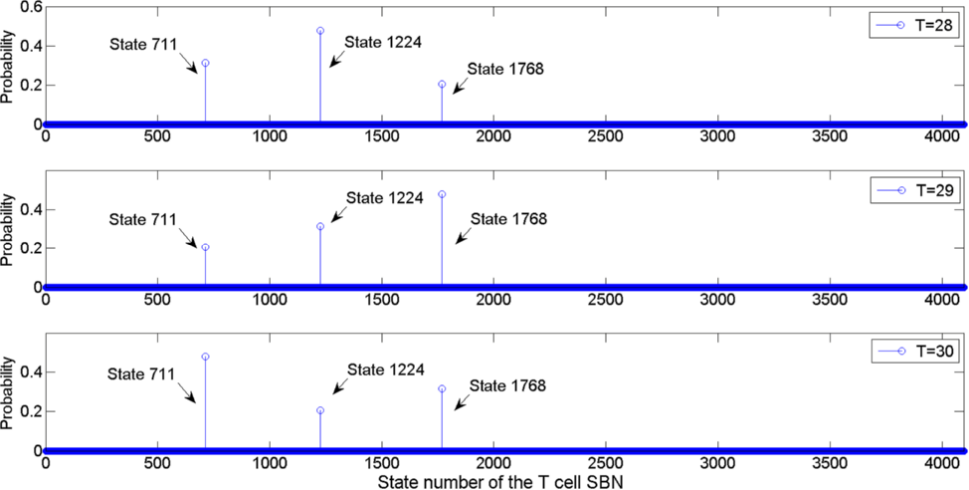
**State distributions of the SBN in Figure**10**after 28, 29 and 30 clock cycles obtained using the time-frame expansion technique.** Our simulation shows that the output distribution starts to oscillate after 20 clock cycles.

Alternatively, and more efficiently, the aforementioned time-frame expansion technique can be used to estimate the attractors with a greatly reduced complexity. The results are shown in Figure 11 for the same SBN simulation of 28, 29 and 30 cycles and the largest runtime is only 0.22s, compared to more than 70s by using the matrix-based analysis. It can be seen that the steady states in Figure 11 match the attractors in Table 9. This shows the effectiveness and efficiency of the time-frame expansion technique.

#### 4. Perturbation and prediction

**Figure 12:**
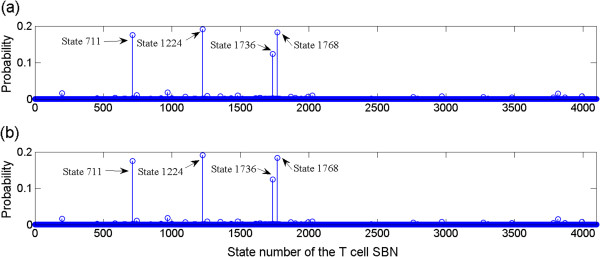
Steady state distribution of the T cell network with perturbation rate of 0.01: (a) computed using state transition matrices and (b) obtained using the time frame expansion technique.

When the genes in a network are perturbed with a small probability, an SBN with perturbation can be constructed (as in Figure 3) for analyzing the stability of the network under perturbation. Since biological networks are usually robust and stable, the same attractors are often expected to be among the steady states with the highest probabilities for the same network by a small perturbation. Assume that each gene is independently perturbed by a probability 0.01, Figure 12(a) shows the steady state distribution of the SBN with perturbation for the network in Figure 9.

It can be seen that the steady states in Figure 12(a) with the highest probabilities 0.1901, 0.1804 and 0.1750 match the known Attractors 1, 2 and 3 (or, states 1224, 1768 and 711). What is interesting, however, is that pseudo-attractors exist in a perturbed network. Pseudo-attractors are the steady states with relatively large probabilities due to random gene perturbation, but they are not the attractors in a network without perturbation. The pseudo-attractors with a steady state probability equal or larger than 0.01 are listed in Table 10. It can be seen that most of these pseudo-attractors differ from the closest known attractor by only one gene. In particular, the most prominent pseudo-attractor, located at state 1736 with a probability larger than 0.1, differs from Attractor 2 or state 1768 by the expression of L-Myb12. L-Myb12 is a late response gene and plays an important role in the regulation of the T-cell network, so this result confirms the sensitivity of L-Myb12 in the regulatory behaviour. Since biological experiments are not straightforward or easy to be implemented for investigating the T-cell network under perturbation, such study may provide insights into the understanding of potential physiological implications in a perturbed network. In a long run, this may be helpful in the development of genetic therapeutic methodologies.

**Table 10:** Pseudo-attractors with a steady state probability no smaller than 0.01, as found in the SBN with perturbation

**State Number**	**Probability**	**Closest attractor**	**Difference**
1736	0.1099	Attractor 2	g(6) (L-Myb12)
967	0.0203	Attractor 3	g(9) (I-Rpol-hnr)
199	0.0164	Attractor 3	g(10) (E-stat5a)
3816	0.0147	Attractor 2	g(12) (L-Mcmd)
3866	0.0135	Different from all the attractors by more than 3 genes	
743	0.0120	Attractor 3	g(6) (L-Myb12)
1352	0.0101	Attractor 1	g(8) (E-Stat1-6)
			g(9) (I-Rpol-hnr)
1256	0.0100	Attractor 1	g(6) (L-Myb12)

(perturbation probability = 0.01; state 1224 with probability 0.1901, state 1768 with probability 0.1804 and state 711 with probability 0.1750).

Application of the time-frame expansion technique yields similar predictions for the network under perturbation. For a perturbation rate of 0.01 and a threshold value of 0.01 for Norm infinity, it only takes 3.7 seconds to obtain the steady state distribution using a sequence length of 10,000 bits, in contrast to 212.1 seconds using the matrix-based SBN method and 2532.9 seconds using the analytical method in [22]. The simulation results are shown in Figure 12(b) for the initial state 1730 (as considered in [43]), which agree with those in Figure 12(a). As the speed of convergence of the time frame expansion technique is dependent on the initial state of the network, several different initial states have been randomly selected and all of them have resulted in a runtime less than 100 seconds. Therefore, the time-frame expansion technique provides a highly efficient tool for analysing the dynamics of a network with (and without) perturbation. See Additional file 6: The Matlab program that evaluates the steady state distribution using the time frame expansion technique for the T-cell genetic network with a perturbation rate of 0.01.

The proposed SBN technique is more efficient than a random sampling approach, due to the use of non-Bernoulli sequences of random permutations of fixed numbers of 1’s and 0’s in the representation of initial probabilities [41]. In Figure S3 of the Additional file 1, it is shown that smaller variations generally result in the state transition matrices computed using the SBN technique compared to those obtained using the Monte Carlo (MC) method. The time-frame expansion technique is also more efficient compared to the Markov Chain Monte Carlo (MCMC) method. In Table S1 of the Additional file 1, it is shown that the time-frame expanded SBN technique converges faster to a steady state than the MCMC method, because it requires a fewer number of clock cycles or time frames to converge and generates less pseudo-random numbers at each time frame. These indicate that the proposed SBN approach is more accurate and more efficient than a simple random sampling approach (such as the MC simulation) in the computation of state transition matrices and the evaluation of steady state distributions.

### Relationship to other GRN models

#### 1. Continuous models

Continuous models based on linear or ordinary differential equations can potentially be implemented using SBNs, provided that the underlying principles of the differential equations can be formulated in state transition matrices. In this case, a network of *n* genes is modelled by:

(12) $$\left[\begin{array}{c} \frac{dg_{1}}{dt}\\ \frac{dg_{2}}{dt}\\ \vdots \\ \frac{dg_{n}}{dt} \end{array}\right]=T\left[\begin{array}{c} g_{1}\\ g_{2}\\ \vdots \\ g_{n} \end{array}\right]\text{,}$$

where *g*_*i*_, (*i =* 1, 2, … *n*), indicates the level of a gene and ***T*** is a matrix of *n* rows and *n* columns. The entries in ***T*** are determined by factors such as the reaction rate constants. If the gene level can be expressed as the occurrence rate of a gene, denoted by *p*_*i*_, (*i =* 1, 2, … *n*), which, for example, can be obtained by the ratio between the number of a particular type of genes and the total number of genes, then (12) can be expressed as:

(13) $$\left[\begin{array}{c} \frac{dp_{1}}{dt}\\ \frac{dp_{2}}{dt}\\ \vdots \\ \frac{dp_{n}}{dt} \end{array}\right]=T\left[\begin{array}{c} p_{1}\\ p_{2}\\ \vdots \\ p_{n} \end{array}\right]\text{.}$$

In an SBN, the next state of genes, ***X***_***t*+1**_, is determined by the current state, ***X***_***t***_, and the state transition matrix, ***A***, i.e.,

(14) $$X_{t+1}=AX_{t}\text{,}$$

where ***A*** is a 2^n^ × 2^n^ matrix, as given by (2). Then a new transition matrix of *n* rows and *n* columns, denoted by *G*, can be obtained by summarizing the entries in the rows and columns of ***A***, such that

(15) $$P_{t+1}=GP_{t}\text{,}$$

where ***P***_***t*+1**_ and ***P***_***t***_ indicate the gene levels at two consecutive time steps. Further assume that

(16) $$\Delta P=P_{t+1}-P_{t}\text{.}$$

In the limit, we obtain:

(17) $$\frac{dP}{dt}=\frac{P_{t+1}-P_{t}}{dt}=\frac{G-I}{dt}P_{t}\text{,}$$

where ***I*** is the identity matrix. Finally, (13) and (17) lead to

(18) G−I=T·dt,

which describes the relationship between the transition matrices in a continuous model and an SBN.

#### 2. Single-molecule level models

In a single-molecule level model, significant stochastic effects of biochemical reactions are accounted for each molecular species. The stochastic simulation algorithm (SSA) tracks the number of molecular species in a biochemical system, so it accurately simulates the discrete, random biochemical reactions specified by the chemical master equation (CME) [9,10]. Essentially, the SSA follows a discrete Markov process, in which two values are generated from two independent random variables at each time step. The first value predicts when the next reaction will occur and the second decides which reaction will occur. In order to characterize the evolution of the system, repeated trials are required to perform, which leads to a significant run time for simulating a large network.

Due to the same underlying Markov models in the SSA and PBNs, the SSA can, in principle, be implemented using SBNs. However, this implementation is not straightforward as the SSA simulates the function of the CME while the SBN implements the state transitions of Boolean functions. A challenge is therefore to formulate the underlying principles of the CME in the form of state transition matrices. Nevertheless, it is possible for the SSA and SBN to be used in a hybrid method. In this method, a logical model is first used to simulate a large network and to identify the sensitive nodes in the network. Then, a single-molecule level model such as the SSA can be used to find out more details of the identified sensitive genes. In this way, this hybrid method leverages the efficiency of a logical model and the accuracy of a single-molecule level model, so it may provide an effective means to model large gene regulatory networks.

### Application on GRN analysis

**Figure 13:**
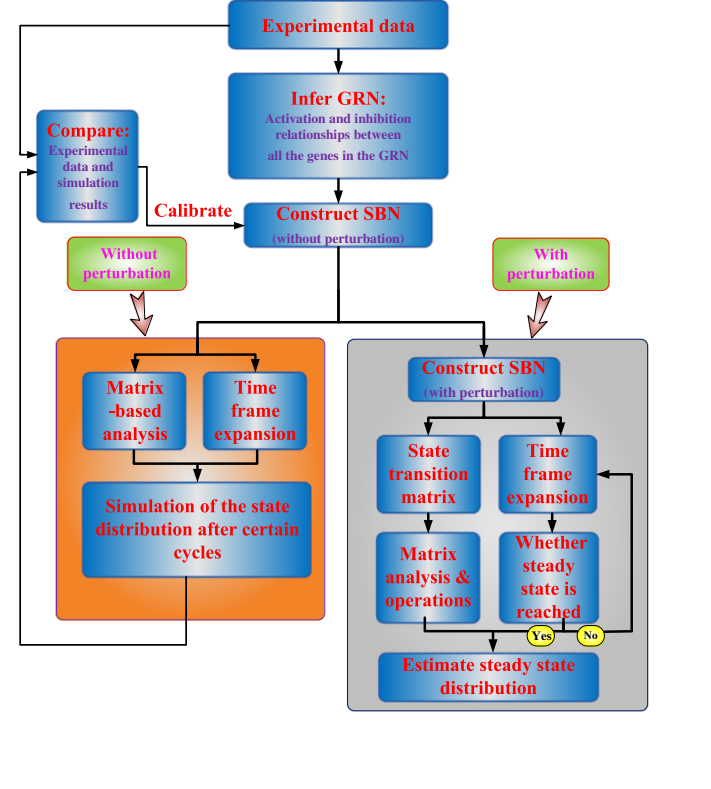
A flowchart for the application of the SBN approach in GRN analysis.

In summary, for a GRN inferred from microarray time series data, an SBN can be constructed to analyze the dynamics of the network with or without gene perturbation. This provides the biologists an efficient tool to evaluate the steady state distribution of a genetic network. A general procedure for applying the proposed SBN approach in a GRN analysis is given in the flowchart of Figure 13. Matlab packages for applications using SBNs, including both for the matrix-based analysis and the time-frame expansion technique, are provided as Additional files.

## Conclusions

This paper proposes a novel structure of stochastic Boolean networks (SBNs) for an accurate and efficient implementation of probabilistic Boolean networks (PBNs). The application of an SBN is demonstrated through the computation of the state transition matrix and the steady-state analysis of a PBN. The state transition matrix can be accurately and efficiently computed in an SBN with a complexity of *O*(*nL*2^*n*^), where *n* is the number of genes in a PBN and *L* is a factor determined by the stochastic sequence length. Since the required minimum sequence length for a given evaluation accuracy usually increases slower with *n* than the number of Boolean networks, i.e., *N*, *L* is typically smaller than *N*, especially in a network with a large number of genes. This result is an improvement compared to the previous results of *O*(*nN*2^2*n*^) and *O*(*nN*2^*n*^). The steady state distribution can be estimated using the obtained state transition matrix or a time-frame expansion technique. The latter approach has shown a significant speedup in the computation of the steady state distribution.

SBNs have been constructed for the p53-Mdm2 network and an inferred T cell immune response network. Simulations of the SBNs have recovered state dynamics that have been experimentally demonstrated for these two networks. The proposed approach is able to discover network dynamics when the genes are under perturbation, which is a difficult task to implement in experiments or by other modeling approaches due to its complexity. So in this case, the SBN technique can be used to provide biologically meaningful insights for a first understanding of the dynamics of a GRN. The relationship between an SBN and continuous/stochastic models has also been discussed and a hybrid approach may be useful in a more efficient modelling of a large GRN. Finally, the SBN approach is able to account for signalling pathway information [48], so it may provide an effective solution to the modeling of complex genetic networks.

## Competing interests

The authors declare that they have no competing interests.

## Authors’ contributions

JL and JH conceived the study and participated in its design. JL carried out the GRN studies, performed the statistical analysis and drafted the manuscript. JH participated in the GRN studies and revised the manuscript. All authors read and approved the final manuscript.

## Supplementary Material

Additional file 1 Stochastic Logic using Non-Bernoulli Sequences. Click here for file

Additional file 2 mux2.m. ‘mux2.m’ is a Matlab program, which implements the function of a two-input stochastic multiplexer (MUX, with one control input) for an SBN. Click here for file

Additional file 3 mux4.m. ‘mux4.m’ is a Matlab program, which implements the function of a four-input stochastic multiplexer (MUX, with two control inputs) for an SBN. Click here for file

Additional file 4 Truth Table of the PBN Inferred from the T Cell Microarray Time Series Data. Click here for file

Additional file 5 **T_cell_SBN.m. ‘T_cell_SBN.m’ is a Matlab program, which describes the structure of an SBN for the T-cell genetic network and computes its state transition matrix for both without and with perturbation.** The programs ‘mux2.m’ and ‘mux4.m’ are needed to run ‘T_cell_SBN.m.’ Click here for file

Additional file 6 **time_frame_expansion.m. ‘time_frame_expansion.m’ is a Matlab program, which evaluates the steady state distribution using the time frame expansion technique for the T-cell genetic network.** The programs ‘mux2.m’ and ‘mux4.m’ are needed to run ‘time_frame_expansion.m.’ Click here for file
