# Supplementary material for: Stochastic Boolean networks: An efficient approach to modeling gene regulatory networks
Source: BMC Syst Biol. 2012 Aug 28;6:113. doi: 10.1186/1752-0509-6-113 (PMC3532238; doi:10.1186/1752-0509-6-113)
Supplement: Additional file 1 — Stochastic Logic using Non-Bernoulli Sequences. [file 1752-0509-6-113-S1.pdf]

Stochastic Boolean Networks: An Efficient Approach to Modeling Gene Regulatory Networks

Jinghang Liang[1], Jie Han[1§]

[1]Department of Electrical and Computer Engineering, University of Alberta, Edmonton, AB, Canada T6G 2V4

[§]Corresponding author

Email addresses:

JL: Jinghang@ualberta.ca

JH: jhan8@ualberta.ca

Additional file 1.

Stochastic Logic using Non-Bernoulli Sequences

1. Stochastic Logic

In stochastic computation, signal probabilities are encoded into binary bit streams, i.e., serially in the time domain. Randomly generated bit streams are used to encode signal probabilities; a specific probability is represented by a number of bits set to a value that is usually in proportion to the mean number of 1's in a bit stream. Fig. S1 shows a stochastic encoding and an inverter. As Boolean operations can be mapped to arithmetic operations, the inverter probabilistically implements a complement operation. Note that in Fig. S1, a sequence length of 10 bits is used for illustration purposes; a larger sequence length is usually needed in practice.

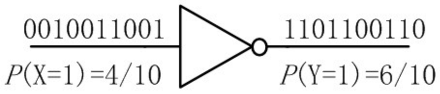

Fig. S1. An inverter and a stochastic encoding.

Stochastic computation transforms Boolean logic operations into probabilistic computations in the real domain. Although each binary bit is processed by a Boolean gate, signal operations are no longer Boolean in nature, but they are arithmetic computations by stochastic logic. Bernoulli sequences are often used as binary bit streams in stochastic computation [1, 2]. In a Bernoulli sequence, every bit is independently generated with a probability p. The mean and variance of the number of 1's in an N-bit Bernoulli sequence are respectively given by

$$\mu = Np, \tag{1}$$

and

$$v = Np(1-p). \quad (2)$$

For the inverter of Fig. S1, if the input probability is a, the mean number of 1's in its output sequence is

$$\mu_1 = N(1-a), \quad (3)$$

and the variance is

$$v_1 = Na(1-a). \quad (4)$$

This is the same as the variance of the input sequence.

Complex arithmetic operations can be implemented by simple stochastic logic. For instance, multiplication can be implemented by an AND gate, as shown in Fig. S2(b). In this multiplication, the input binary streams must not be correlated for a correct computation. However, the bit-wise dependencies between the input random binary streams can be used to yield new stochastic logic models that account for the statistical correlation in input signals. This is shown in Fig. S2(a) as a general stochastic model of AND in which the two input signals may be correlated.

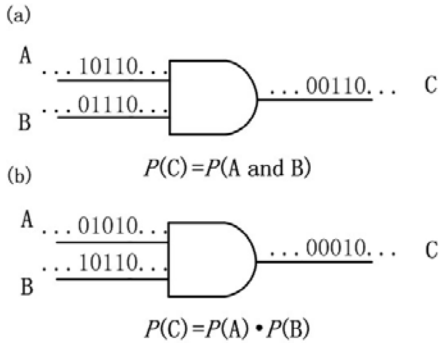

Fig. S2. Stochastic AND logic: (a) the general model; (b) the special case of multiplication, when the two inputs are statistically independent.

If the inputs of the AND are two independent Bernoulli sequences with generating probabilities *a* and *b* respectively, the mean number of 1's in the output sequence is:

$$\mu_2 = Nab, \quad (5)$$

and the variance is given by:

$$v_2 = Nab(1 - ab). \tag{6}$$

For the AND gate in Fig. S2(a) with possibly correlated inputs,

$$P(C = 1) = P(A = 1, B = 1) = P(A = 1)P(B = 1|A = 1). \tag{7}$$

Let $a = P(A = 1)$, $b = P(B = 1)$ and $p_c = P(B = 1|A = 1)$; then

$$P(C = 1) = ap_c. \tag{8}$$

The use of Bernoulli sequences as inputs results in a Bernoulli sequence at the output with a generating probability given by (8); therefore, the mean number of 1's in the output sequence and its variance are given by:

$$\mu_{2,g} = Nap_c, \tag{9}$$

and

$$v_{2,g} = Nap_c(1 - ap_c), \tag{10}$$

respectively.

The use of Bernoulli sequences however incurs a large computational overhead that severely limits its application for an efficient analysis. This aspect is addressed through the use of non-Bernoulli sequences, as discussed next.

2. Non-Bernoulli Sequences

In this work, non-Bernoulli sequences are used for reducing the computational complexity and inaccuracy. Specifically, each initial input stochastic sequence contains a fixed number of 1's and the positions of the 1's are determined by a random permutation. For a given probability *p* and a sequence length of *N* bits, the number of 1's to be generated is given by *Np*. The output distributions of the inverter and AND gate when non-Bernoulli sequences are used as inputs, are treated in more detail next.

For an inverter, assume that the input has a probability of *a* to be “1”; so *Na* is the number of 1’s in the input sequence of *N* bits. Then the expected value of 1’s in the output sequence is given by:

$$\mu_1' = N(1-a). \qquad (11)$$

Since there is no variation in the input, the variance in the output is considered to be 0, i.e.,

$$v_1' = 0. \qquad (12)$$

For an AND gate, the use of the non-Bernoulli sequences resembles von Neumann’s NAND multiplexing technique, as discussed in [3, 4] for fault-tolerant logic design. The following Lemma shows that its output follows approximately a Gaussian distribution when the sequence length *N* is large.

Lemma 1: For an AND gate, assume that the two inputs are “1” with probabilities *a* and *b* and represented by non-Bernoulli sequences of *N* bits (as random permutations of fixed numbers of 1’s and 0’s). For a large *N*, the output sequence follows a Gaussian distribution with a mean number of 1’s given by:

$$\mu_2' = Nab, \qquad (13)$$

and a variance:

$$v_2' = Na(1-a)b(1-b). \qquad (14)$$

Proof: The two input probabilities *a* and *b* give $r=aN$ and $s=bN$ as the numbers of 1’s in the input sequences. In these two inputs, the numbers of possible permutations are:

$$C_a = \binom{N}{r} = \frac{N!}{r!*(N-r)!}, \qquad (15)$$

and

$$C_b = \binom{N}{s} = \frac{N!}{s!*(N-s)!}, \qquad (16)$$

respectively. Assume that the AND gate produces t 1's in the output sequence; then, the number of permutations that causes this occurrence, can be obtained by combinatorial analysis [3, 4]. This leads to:

$$C_o = \binom{N}{t} * \binom{N-t}{r-t} * \binom{N-r}{s-t} = \frac{N!}{t! * (r-t)!(s-t)!(N-r-s+t)!} \quad (17)$$

The probability that t 1's result in the output sequence, is given by the number of output permutations divided by the total possible number of input permutations, i.e.,

$$P(t) = \frac{C_o}{C_a * C_b} = \frac{r!(N-r)!s!(N-s)!}{t!(r-t)!(s-t)!(N-r-s+t)!N!}. \quad (18)$$

Assume that the expected output probability is z, and therefore

$$z = \frac{t}{N}. \quad (19)$$

As per [3], the application of Stirling's formula results in:

$$P(z) \sim \frac{1}{\sqrt{2\pi N}} \sqrt{\beta} e^{-\theta N}, \quad (20)$$

where

$$\beta \sim \frac{1}{a(1-a)b(1-b)}, \quad (21)$$

$$\theta \sim \frac{(z-ab)^2}{2a(1-a)b(1-b)}. \quad (22)$$

(20), (21) and (22) indicate that the output sequence follows approximately a Gaussian distribution with a mean number of 1's given by (13) and a variance given by (14). □

3. Non-Bernoulli vs. Bernoulli Sequences

Next, the comparison between the use of Bernoulli and non-Bernoulli input sequences in stochastic logic is pursued. For an inverter, it is easy to find that (11) = (3) and (12) = 0. This indicates that the use of non-Bernoulli input sequences results in a deterministic output value equal to the mean value of the one by using Bernoulli input sequences. For an AND gate, the following theorem applies for independent inputs.

Theorem 1: Compared to the case when Bernoulli sequences are used to represent the initial input probabilities, the use of large non-Bernoulli sequences as random permutations of fixed numbers of 1's and 0's results in an output sequence with the same mean number of 1's and a smaller variance for an AND gate when its inputs are independent.

Proof: From Lemma 1, it can be seen that (13) = (5) and

$$v_2 - v_2' = Nab(1-ab) - Na(1-a)b(1-b) = Nab(a(1-b) + b(1-a)) \geq 0, \tag{23}$$

so proving the theorem. □

The general case of correlated inputs is considered as follows. When non-Bernoulli sequences are used as inputs, the random permutation allows for some randomness in the inputs, albeit with a correlation between them. Without loss of generality, assume that input *A* is first generated; input *B* is then generated conditionally on *A*. For the 1's in the sequence of *A*, further assume that the corresponding bits in *B* are generated as a Bernoulli sequence with probability p_c. For the 0's in the sequence of *A*, subsequently, the number of 1' in the corresponding bits in *B* is actually determined due to the nature of the non-Bernoulli sequence used to represent input *B*. Since the number of 1's in the sequence of *A* is *Na*, the mean number of 1's in the corresponding bits in *B* and its variance are given by:

$$\mu_{2,g}' = Nap_c, \tag{24}$$

and

$$v_{2,g}' = Nap_c(1-p_c). \tag{25}$$

The combinations of 1's in inputs *A* and *B* produce the 1's in the output sequence, so the mean number of 1's at the output and the variance are given by (24) and (25)

respectively for an AND gate with non-Bernoulli input sequences that may be correlated.

Hence, it can be seen that (9) = (24) and from (10) and (25),

$$v_{2,g} - v'_{2,g} = Nap_c(1 - ap_c) - Nap_c(1 - p_c) = Nap_c{}^2(1 - p_c) \geq 0. \quad (26)$$

This indicates that, when compared to Bernoulli input sequences, the use of non-Bernoulli input sequences as random permutations of fixed numbers of 1's and 0's results in an output sequence with the same mean number of 1's and a smaller variance for an AND gate when its inputs may be correlated.

Any logic function can be implemented with inverters and AND gates; so, a smaller variance in the output of AND gates (as achieved by using the non-Bernoulli inputs) will result in a smaller variance in the output of a function implemented with inverters and AND gates. Also, the same mean value results from the use of non-Bernoulli and Bernoulli inputs. Therefore in a logic network, the use of non-Bernoulli and Bernoulli sequences as initial inputs will produce evaluation results with the same mean, but different variance; the former method results in a smaller variance than the latter method.

We conjecture this result as follows: compared to the case when Bernoulli sequences are used to represent the initial input probabilities, the use of large non-Bernoulli sequences as random permutations of fixed numbers of 1's and 0's results in an output sequence with the same mean number of 1's and a smaller variance for a combinational logic network.

4. Comparison of the SBN and Monte Carlo (MC) Methods

The SBN and Monte Carlo (MC) methods are first used to compute the state transition matrices for a randomly-generated 5-gene network. The obtained matrices ($\boldsymbol{A_{SBN}}$ and $\boldsymbol{A_{MC}}$) are then compared to the result obtained using the analytical approach in [5]

$(\boldsymbol{A_{GRN}})$. Fig. S3 shows the accuracies of $\boldsymbol{A_{SBN}}$ and $\boldsymbol{A_{MC}}$ against $\boldsymbol{A_{GRN}}$, given by the measures of norm 1, norm 2 and norm infinity of $(\boldsymbol{A_{GRN}} - \boldsymbol{A_{SBN}})$ and $(\boldsymbol{A_{GRN}} - \boldsymbol{A_{MC}})$. In Fig. S3, (a), (b) and (c) show the simulation results of 1000 SBN evaluations with a sequence length of 10000 binary bits, while (d), (e) and (f) show the simulation results of 1000 MC experiments of each containing 10000 simulations.

It can be seen that the variations are smaller in the state transition matrices computed using the SBN technique than in those obtained using the MC method, indicated by a better proximity of the SBN results to the analytical result measured in all three norms.

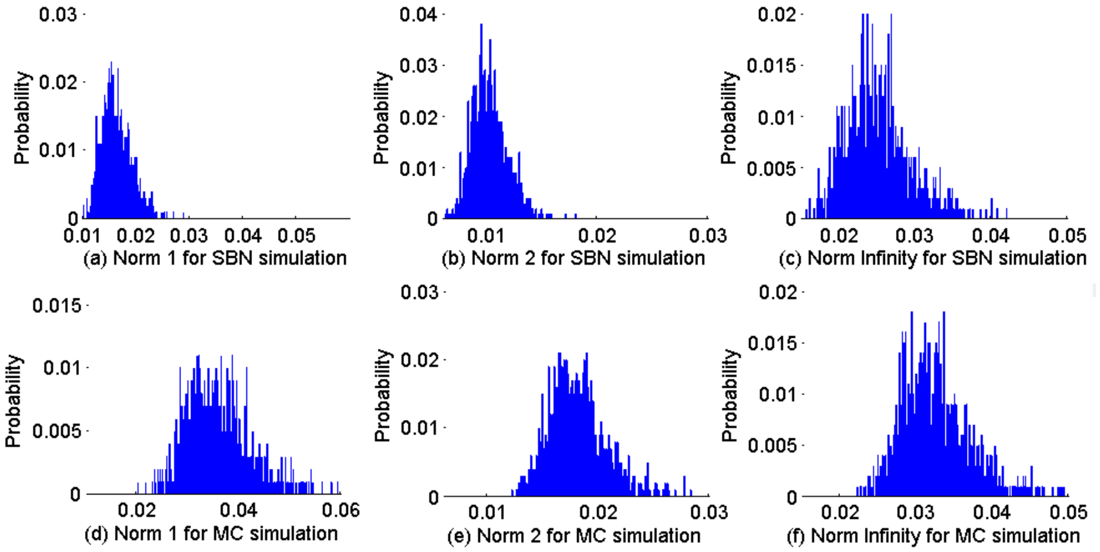

Fig. S3. Probability distribution histograms of the SBN and MC methods, measured by different norms of $(A_{GRN} - A_{SBN})$ and $(A_{GRN} - A_{MC})$. Using approximations of Gaussian distributions, the mean values and standard deviations are (a) μ_a = 0.0162, v_a = 0.0027, (b) μ_b = 0.0103, v_b = 0.0016, (c) μ_c = 0.0252, v_c = 0.0042, (d) μ_d = 0.0359, v_d = 0.0061, (e) μ_e = 0.0180, v_e = 0.0026, (f) μ_f = 0.0325, v_f = 0.0046.

The time-frame expanded SBN technique resembles the Markov Chain Monte Carlo (MCMC) method in the sense that both methods use random vectors in the simulation. In Table S1, the runtime for the T cell network [6] is shown for the time-frame

expanded SBN technique to obtain a steady state, compared to the MCMC method. Due to the use of non-Bernoulli sequences of random permutations of fixed numbers of 1's and 0's, the SBN requires fewer pseudo-random number generations at each time frame and the computed matrix at each time frame is more accurate (as shown in Fig. S3). Therefore, the state of a network converges faster to a steady state in a time-frame expanded SBN than using the MCMC method. These results indicate that the proposed SBN approach is more accurate and more efficient than a random sampling based method (such as the MC simulation) in the computation of state transition matrices and the evaluation of steady state distributions.

Table S1. Time consumption of the time-frame expanded SBN technique for the T cell network for different threshold values, compared to the MCMC method.

Threshold value (norm 2)	Sequence length (bits)	Perturbation rate	SBN		MCMC	
			No. of convergence cycles	Time (s)	No. of convergence cycles	Time (s)
0.001	100,000	0.0001	84	56.178200	196	149.218028
		0.001	59	39.673542	106	80.726064
		0.01	34	23.693967	49	37.285877
0.01	10,000	0.01	13	0.897627	14	1.111401
		0.001	24	1.521310	26	2.028786
		0.0001	31	1.947808	30	3.515963

References

1. B. R. Gaines, **Stochastic Computing Systems**, *Advances in Information Systems Science*, Vol. 2, pp. 37-172, 1969.
2. B. Brown and H. Card, **Stochastic neural computation I: Computational elements,** *IEEE Tran. Computers*, vol. 50, pp. 891–905, Sept. 2001.
3. J. von Neumann, **Probabilistic logics and the synthesis of reliable organisms from unreliable components**, *Automata Studies, Shannon C.E. & McCarthy J., eds., Princeton University Press*, pp. 43-98, 1956.
4. Jie Han, **Fault-Tolerant Architectures for Nanoelectronic and Quantum Devices**, *Universal Press*, Veenendaal, The Netherlands, 2004. A Ph.D.

dissertation of the Delft University of Technology, 1-135. ISBN: 90-9018888-6

5. Zhang, S. et al: **Simulation study in probabilistic Boolean network models for genetic regulatory networks.** *Int. J. Data Min.* 2007, 1:217-240.
6. Martin, S., Zhang, Z., Martino, A. and Faulon, J-L.: **Boolean dynamics of genetic regulatory networks inferred from microarray time series data.** *Bioinformatics*, 2007, 23(7): 866-874.
