# Supplementary material for: Stochastic Boolean networks: An efficient approach to modeling gene regulatory networks
Source: BMC Syst Biol. 2012 Aug 28;6:113. doi: 10.1186/1752-0509-6-113 (PMC3532238; doi:10.1186/1752-0509-6-113)
Supplement: Additional file 4 — Truth Table of the PBN Inferred from the T Cell Microarray Time Series Data. [file 1752-0509-6-113-S4.pdf]

Stochastic Boolean Networks: An Efficient Approach to Modeling Gene Regulatory Networks

Jinghang Liang[1], Jie Han[1§]

[1]Department of Electrical and Computer Engineering, University of Alberta,

Edmonton, AB, Canada T6G 2V4

[§]Corresponding author

Email addresses:

JL: Jinghang@ualberta.ca

JH: jhan8@ualberta.ca

Additional file 2.

Truth Table of the PBN Inferred from the T Cell Microarray Time Series Data

Based on the activation and inhibition relationships in the T cell network in Fig. 9 [1], the following single-input Boolean functions are obtained using the proposed SBN rules:

$$\mathbf{g_1} = not(\mathbf{g_6}),$$

$$\mathbf{g_3} = \mathbf{g_2},$$

$$\mathbf{g_4} = not(\mathbf{g_2}),$$

$$\mathbf{g_5} = not(\mathbf{g_3}),$$

$$\mathbf{g_7} = \mathbf{g_3},$$

$$\mathbf{g_8} = not(\mathbf{g_5}),$$

$$\mathbf{g_{10}} = \mathbf{g_{11}},$$

$$\mathbf{g_{12}} = not(\mathbf{g_8}).$$

For $\mathbf{g_2}$, $\mathbf{g_6}$, $\mathbf{g_9}$ and $\mathbf{g_{11}}$, their Boolean functions are determined by multiple inputs, as shown as follows.

1. The state of $\mathbf{g_2}$ is determined by the states of three genes: $\mathbf{g_2}$, $\mathbf{g_3}$ and $\mathbf{g_6}$.

Truth Table for $\mathbf{g_2}$: $\mathbf{f_1^{(2)}} = \mathrm{or}(\mathbf{g_2}, \mathbf{g_6})$, $\mathbf{f_2^{(2)}} = \mathrm{or}(\mathbf{g_3}, \mathbf{g_6})$.

$\mathbf{g_2 g_3 g_6}$	$\mathbf{f_1^{(2)}}$	$\mathbf{f_2^{(2)}}$
000	0	0
001	1	1
010	0	1
011	1	1
100	1	0
101	1	1
110	1	1
111	1	1
$c_j^{(i)}$	0.5	0.5

2. The state of $\mathbf{g_6}$ is determined by five genes: $\mathbf{g_1, g_9, g_{11}, g_{12}}$ and $\mathbf{g_{10}}$.

Truth Table for $\mathbf{g_6}$: $\mathbf{f_1^{(6)}}$ = and(or($\mathbf{g_1, g_9, g_{11}, g_{12}}$), not($\mathbf{g_{10}}$)), $\mathbf{f_2^{(6)}}$ = not($\mathbf{g_{10}}$).

$\mathbf{g_{10} g_1 g_9 g_{11} g_{12}}$	$\mathbf{f_1^{(6)}}$	$\mathbf{f_2^{(6)}}$
00000	0	1
00001	1	1
00010	1	1
00011	1	1
00100	1	1
00101	1	1
00110	1	1
00111	1	1
01000	1	1
01001	1	1
01010	1	1
01011	1	1
01100	1	1
01101	1	1
01110	1	1
01111	1	1
10000	0	0
10001	0	0
10010	0	0
10011	0	0
10100	0	0
10101	0	0
10110	0	0
10111	0	0
11000	0	0
11001	0	0
11010	0	0
11011	0	0
11100	0	0
11101	0	0
11110	0	0
11111	0	0
$c_j^{(i)}$	0.8	0.2

3. The state of g_9 is determined by the states of three genes: g_4, g_5 and g_{12}.

Truth table for g_9: $f_1^{(9)} = or(g_4, g_{12})$, $f_2^{(9)} = or(g_5, g_{12})$.

$g_4 g_5 g_{12}$	$f_1^{(9)}$	$f_2^{(9)}$
000	0	0
001	1	1
010	0	1
011	1	1
100	1	0
101	1	1
110	1	1
111	1	1
$c_j^{(i)}$	0.5	0.5

4. The state of $\mathbf{g_{11}}$ is determined by the states of four genes, $\mathbf{g_6, g_4, g_5}$ and $\mathbf{g_7}$.

Truth table for $\mathbf{g_{11}}$: $\mathbf{f_1^{(11)}} = \mathrm{and}(\mathrm{or}(\mathbf{g_7}, \mathrm{nor}(\mathbf{g_4}, \mathbf{g_5})), \mathrm{not}(\mathbf{g_6}))$,

$\mathbf{f_2^{(11)}} = \mathrm{and}(\mathrm{nor}(\mathbf{g_4}, \mathbf{g_5}), \mathrm{not}(\mathbf{g_6}))$,

$\mathbf{f_3^{(11)}} = \mathrm{and}(\mathbf{g_7}, \mathrm{not}(\mathbf{g_6}))$, $\mathbf{f_4^{(11)}} = \mathrm{not}(\mathbf{g_6})$.

$\mathbf{g_6 g_4 g_5 g_7}$	$\mathbf{f_1^{(11)}}$	$\mathbf{f_2^{(11)}}$	$\mathbf{f_3^{(11)}}$	$\mathbf{f_4^{(11)}}$
0000	1	1	0	1
0001	1	1	1	1
0010	0	0	0	1
0011	1	0	1	1
0100	0	0	0	1
0101	1	0	1	1
0110	0	0	0	1
0111	1	0	1	1
1000	0	0	0	0
1001	0	0	0	0
1010	0	0	0	0
1011	0	0	0	0
1100	0	0	0	0
1101	0	0	0	0
1110	0	0	0	0
1111	0	0	0	0
$c_j^{(i)}$	0.185	0.37	0.148	0.297

References

1. Martin, S., Zhang, Z., Martino, A. and Faulon, J-L.: **Boolean dynamics of genetic regulatory networks inferred from microarray time series data.** *Bioinformatics*, 2007, 23(7): 866-874.
